# Pharmacokinetic and Pharmacodynamic Evaluation of PZ-2891, an Anti-Alzheimer’s Disease Agonist of PANK2

**DOI:** 10.3390/ph18121871

**Published:** 2025-12-09

**Authors:** Ying Chen, Huimin Ma, Mengyao Jin, Shize Zhang, Shimeng Qu, Guangji Wang, Jiye Aa

**Affiliations:** Key Laboratory of Drug Metabolism and Pharmacokinetics, State Key Laboratory of Natural Medicines, China Pharmaceutical University, Nanjing 210009, China; 3122074252@stu.cpu.edu.cn (Y.C.); 3323071889@stu.cpu.edu.cn (H.M.);

**Keywords:** Alzheimer’s disease (AD), Pharmacokinetics (PK), blood–brain barrier (BBB), Pantothenate Kinase 2 (PANK2), oxidative stress, neuroprotector

## Abstract

**Background/Objectives:** Alzheimer’s disease (AD) is a neurodegenerative disorder with a high incidence but limited agents. Herein, **PZ-2891** was discovered as a novel anti-AD candidate. Both in vivo and in vitro pharmacodynamic (PD) studies and pharmacokinetic (PK) properties were investigated and illustrated in this research. **Methods**: A computer-generated random number table was used to divide mice into various groups randomly. Injecting Aβ into the mice hippocampus to mimic AD-like pathologies, neurobehavioral tests, including the Morris maze, Y maze, open field test (OFT) and novel object recognition (NOR), were operated to evaluate the cognitive improvement in **PZ-2891**. D-galactose (D-gal), okadaic acid (OA) and lipopolysaccharide (LPS) were employed to trigger neural injuries in vitro. A reliable analytic method was developed to profile **PZ-2891**’s PK properties in SD rats through a triple quadrupole liquid chromatography–mass spectrometry (LC–MS/MS) instrument. **Results: PZ-2891** markedly alleviated cognitive impairment in the Aβ-induced model mice. It also protected nerve cells from oxidative stress and inflammatory injuries and significantly reduced AD-typical pathological biomarkers. The PK results showed that **PZ-2891** was exposed rapidly in both plasma and brain, with a brain-to-blood ratio of around 0.59, C_max_ of around 454.50 ± 151.35 ng/mL, T_max_ of around 0.49 ± 0.15 h and oral bioavailability of around 19.74 ± 6.78%. **Conclusions:** These findings suggest that **PZ-2891**, an agonist of PANK2, is a novel and potential candidate agent for AD with excellent efficacy and PK properties.

## 1. Introduction

Alzheimer’s disease (AD) is a common neurodegenerative disease, and its incidence increases with age. The clinical manifestations of AD mainly include cognitive decline, memory loss, motor dysfunction and mental and emotional comorbidities [1,2]. According to Alzheimer’s Disease International (ADI), it is estimated that the number of global dementia patients will increase to 139 million by 2050. Currently, the worldwide population is increasingly aging, and AD has become a global social and medical issue [3]. The pathogenesis of AD is complex and involves numerous factors, such as genetics, aging, metabolism and immunity, including amyloid-β (Aβ) protein deposition, excessive phosphorylation of tau protein [4], dysfunction of cholinergic neurons [5], metal ion deposition [6], mitochondrial dysfunction, neuroinflammation and oxidative stress [7]. The development of drugs for AD remains a difficult challenge due to its hidden, protracted and complex course.

Initially, **PZ-2891** was designed as a pantothenate kinase (PANK) modulator [8,9,10], and showed great potential in the treatment of pantothenate kinase-associated neurodegeneration (PKAN) through improving the activity of PANK and the level of coenzyme A (CoA) in neurons [11]. PKAN is an autosomal recessive neurodegenerative disorder related to PANK2 mutation that blocks the CoA biosynthesis pathway, thereby causing a significant decrease in CoA levels in the brain [12,13]. CoA is a key cofactor for energy metabolism, lipid synthesis and maintenance of mitochondrial function, the deficiency of which could induce mitochondrial dysfunction, oxidative stress and iron metabolism imbalance, ultimately leading to neuronal degeneration. It was reported that pantothenic acid, as the necessary precursor for CoA synthesis, is significantly reduced in AD brains, and the acetyl-CoA content of the hippocampus decreases in a dependent manner with age [14]. This not only inhibits neuronal synaptic transmission but also further exacerbates mitochondrial dysfunction. There may be a similar mechanism between PKAN and AD neurodegenerative diseases. In addition, PKAN patients exhibit some clinical symptoms similar to those of AD, including loss of motor function, abnormal muscle tone, a decline in cognitive ability and even dementia. In addition, neuropathology suggests that the aggregation of α-synuclein protein to form Lewy bodies and the accumulation of tau protein to form NFTs could be observed in some brain regions of PKAN patients [15,16].

Given the similar clinical symptoms and pathological features of PKAN and AD [17,18], this study focused on exploring whether **PZ-2891** could play a positive role in AD treatment. On the other hand, although the efficacy of **PZ-2891** on PKAN has been investigated, its PK properties have not yet been fully clarified. Thus, a comprehensive detection method for **PZ-2891** and its main PK parameters was established and investigated in this research to provide a basis and reference for the development and application of PANK regulators.

## 2. Results

### 2.1. Oral Safety Evaluation of PZ-2891

Before the pharmacodynamics studies were performed, a primary assessment of **PZ-2891** safety was conducted to determine a suitable dosage. No death, weight loss or obvious abnormal behavior was observed in C57 mice treated with **PZ-2891** (30 mg/kg) for 14 consecutive days [11]. In addition to general clinical observation, the HE staining results of the liver and kidney also showed the primary safety of **PZ-2891** (Supplementary Figure S1A). The weight indices of main organs (liver, heart, kidney and spleen), as well as alanine aminotransferase (ALT), aspartate aminotransferase (AST), blood urea nitrogen (BUN) and creatinine (CRE) levels, also showed no significant changes between **PZ-2891**-treated and control mice (Supplementary Figure S1B,C).

### 2.2. Behavioral Experiments of PZ-2891 In Vivo

**Figure 1:**
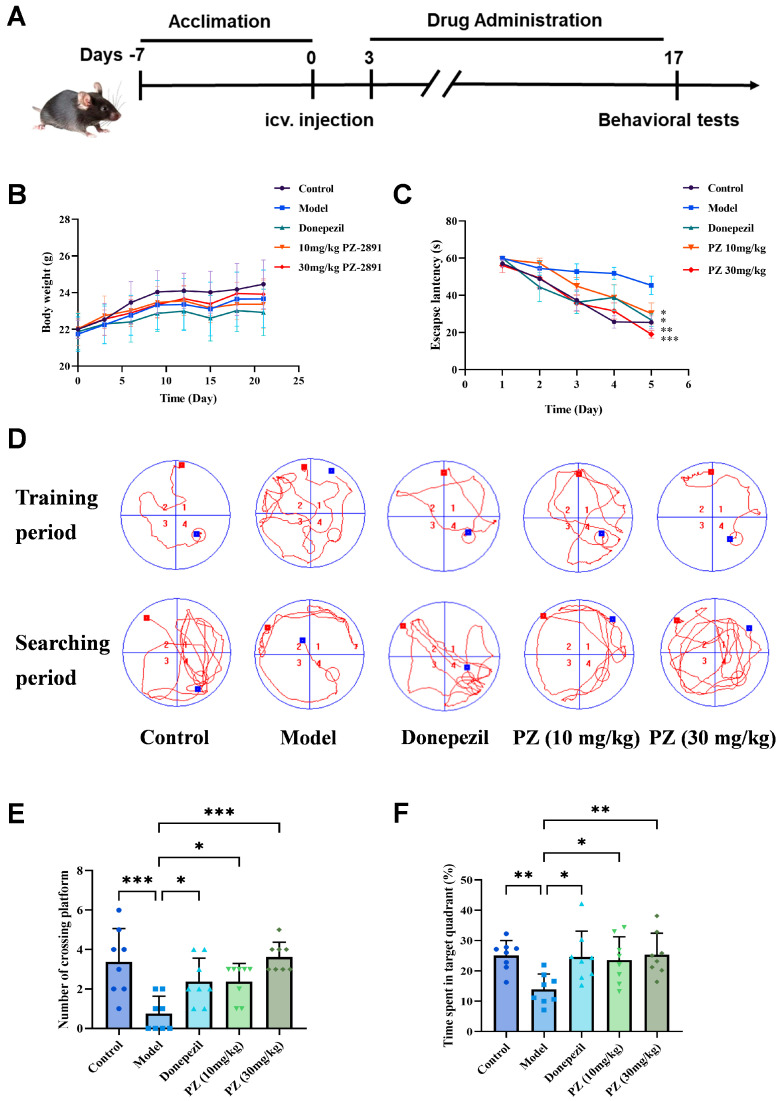
**PZ-2891** improved cognitive ability in the Morris water maze. (**A**) Experimental schedule. (**B**) Weight records of mice from different groups (*n* = 8). (**C**) Escape latency to reach the platform in the maze (*n* = 8). (**D**) Average swimming trajectories of the mice during the training and search periods (*n* = 8). (**E**) The number of platform crossings during the search period (*n* = 8). (**F**) Time spent in the target quadrant during the search period (*n* = 8). The data are expressed as the mean ± SD. *p* values were determined by one-way ANOVA. * *p* < 0.05, ** *p* < 0.01, *** *p* < 0.001.

To mimic pathological AD conditions, Aβ_1-42_ peptides were injected into the hippocampal region to induce cognitive impairment (Figure 1A). The Morris water maze (MWM) could reveal the spatial learning and memory of animals. Compared with control mice, the model mice showed a longer escape latency and a lower number of mice crossing the platform, indicating that injecting Aβ_1-42_ successfully caused cognitive impairment. After treatment with donepezil (5 mg/kg) or **PZ-2891** (10/30 mg/kg), mice exhibited a shorter escape latency and more concise trajectories, and the number of platform crossings and time spent in the target quadrant also increased significantly (Figure 1B–F). A comparison of the low dose and high dose groups revealed that the efficacy of **PZ-2891** was dose-dependent.

**Figure 2:**
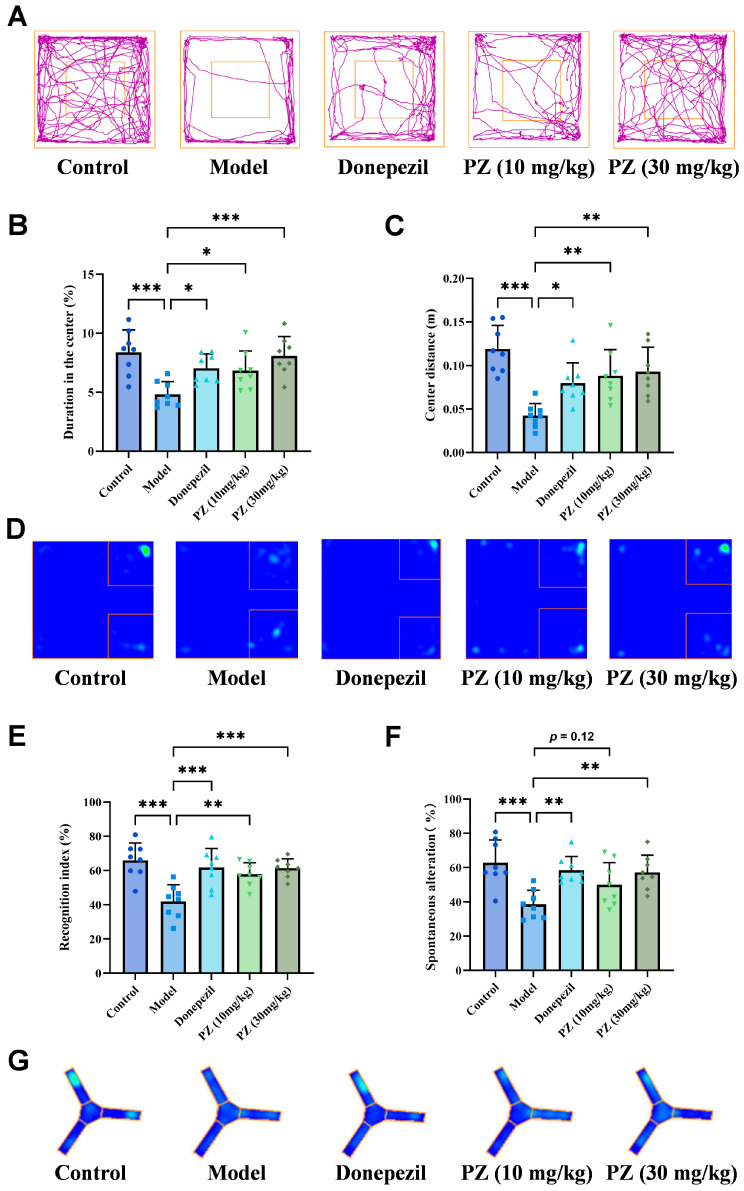
**PZ-2891** functioned as a cognitive improver in the OFT, NOR and Y maze. (**A**) Average movement trajectories of mice in the OFT, the purple lines represented the movement trajectories (*n* = 8). (**B**) Time spent in the central area in the OFT (*n* = 8). (**C**) Movement distance in the central area in the OFT (*n* = 8). (**D**) Average heatmap of mice exploring novel objects. (**E**) Recognition index of the novel object (*n* = 8). (**F**) Spontaneous alternation of the Y maze (*n* = 8). (**G**) Average heatmap of mice exploring the new arm (*n* = 8). The data are expressed as the mean ± SD. *p* values were determined by one-way ANOVA. * *p* < 0.05, ** *p* < 0.01, *** *p* < 0.001.

The OFT results revealed locomotion and anxiety of the animals. The model mice spent less time and crossed a shorter distance in the central zone (Figure 2A–C), suggesting a decline in autonomous movement ability and enhanced anxiety-like symptoms. However, time and distance in the central area were elevated significantly after treating with **PZ-2891**, indicating that it improved the autonomous movement ability and anxiety-like behavior of AD mice.

As shown in the NOR test (Figure 2D,E), the model mice exhibited a decreased recognition index of novel objects, whereas the preference for new objects significantly increased for the mice treated with donepezil and **PZ-2891**, suggesting an improvement in recognition memory.

Similar phenomena were observed in the Y maze, which reflects the working memory of the animals. Model mice exhibited decreased spontaneous alternation and recognition rates in the new arm, suggesting cognitive impairment and spatial memory decline. This effect was reversed after administration with donepezil or **PZ-2891**, with significant increases in alternation and novel arm recognition (Figure 2F,G). Taken together, the behavioral tests indicated that **PZ-2891** could alleviate the cognitive impairment induced by Aβ_1-42_ in C57 mice, and its efficacy was comparable to that of donepezil.

### 2.3. Neuroprotective Effects of PZ-2891 In Vitro

**Figure 3:**
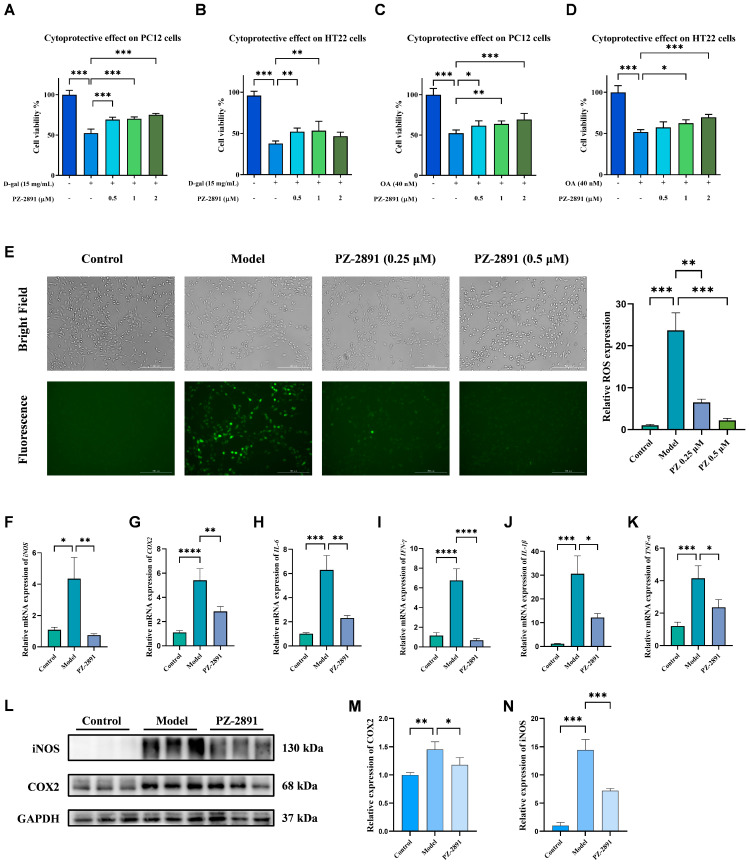
**PZ-2891** functioned as a neuroprotector of oxidative stress and inflammatory reactions induced by D-gal (15 mg/mL) and OA (40 nM). (**A**) Protective effects against D-gal-induced PC12 cell damage (*n* = 6). (**B**) Protective effects against D-gal-induced damage to HT22 cells (*n* = 6). (**C**) Protective effects against OA-induced PC12 cell damage (*n* = 6). (**D**) Protective effects against OA-induced damage to HT22 cells (*n* = 6). (**E**) Determination of ROS in PC12 cells triggered by D-gal, reflected by fluorescence intensity (*n* = 3). (**F**–**K**) Relative mRNA levels of *iNOS*, *COX*-*2*, *IL*-*6*, *IFN*-*γ*, *IL*-*1β* and *TNF*-*α* in BV2 cells induced by LPS (*n* = 8). (**L**) WB results of iNOS and COX-2 in BV2 cells induced by LPS (1 µg/mL) (*n* = 3). (**M**,**N**) Quantitative results of iNOS and COX-2 expression (*n* = 3). The data are expressed as the mean ± SD. *p* values were determined by one-way ANOVA. * *p* < 0.05, ** *p* < 0.01, *** *p* < 0.001, **** *p* < 0.0001.

The beneficial effects of **PZ-2891** in vitro were also demonstrated in this study. After D-gal intervention [19], the survival rates of PC12 and HT22 cells decreased to 52% and 38% of those in the blank group, respectively (Figure 3A,B). In contrast, the viability recovered to approximately 69% and 52% after coincubation with 0.5 µM **PZ-2891**. In addition, OA can cause cell damage due to its strong neurotoxicity [20]. The viability of cells treated with OA was reduced to approximately 50% of that of the blank group, which could be reversed by coincubation with **PZ-2891** (Figure 3C,D). Compared with the model group, cells treated with **PZ-2891** exhibited higher survival rates, and its protective effect occurred in a dose-dependent manner.

The level of ROS was detected to evaluate the cell status after modeling and treatment. As shown in Figure 3E, a stronger green fluorescence intensity (GFI) and decreased cell viability were observed in the model group treated with D-gal, which could cause ROS overproduction and oxidative stress, and thereby cell damage and death. In contrast, the GFI was weakened in the **PZ-2891**-treated group, the number of cells increased in the same area and the level of ROS was reduced by 73% relative to the model mice (*p* < 0.05), suggesting that **PZ-2891** could reduce oxidative stress and protect cells against excess ROS triggered by D-gal.

In addition to oxidative stress’s ability to arouse cell morphological changes, this is also usually accompanied by neuroinflammation, an important pathogenic factor of neurodegenerative diseases [21]. LPS was used to induce BV2 cells to establish a model of neural inflammatory injury. The RT–qPCR results revealed that LPS stimulation significantly triggered the release of proinflammatory cytokines, including inducible nitric oxide synthase (iNOS), cyclooxygenase-2 (COX-2), interleukin-6 (IL-6), interferon-γ (IFN-γ), interleukin-1β (IL-1β) and tumor necrosis factor-α (TNF-α) [22]. Coincubation with **PZ-2891** alleviated the inflammatory reaction and reduced the secretion of cytokines (Figure 3F–K). The WB results also confirmed that LPS stimulation increased the expression of iNOS and COX-2, whereas treatment with **PZ-2891** significantly reversed these effects (Figure 3L–N).

### 2.4. AD Pathology Improvement by PZ-2891 via Upregulation of PANK2 Expression

#### 2.4.1. Pathological Damage Improvement

##### Neuronal Morphology

**Figure 4:**
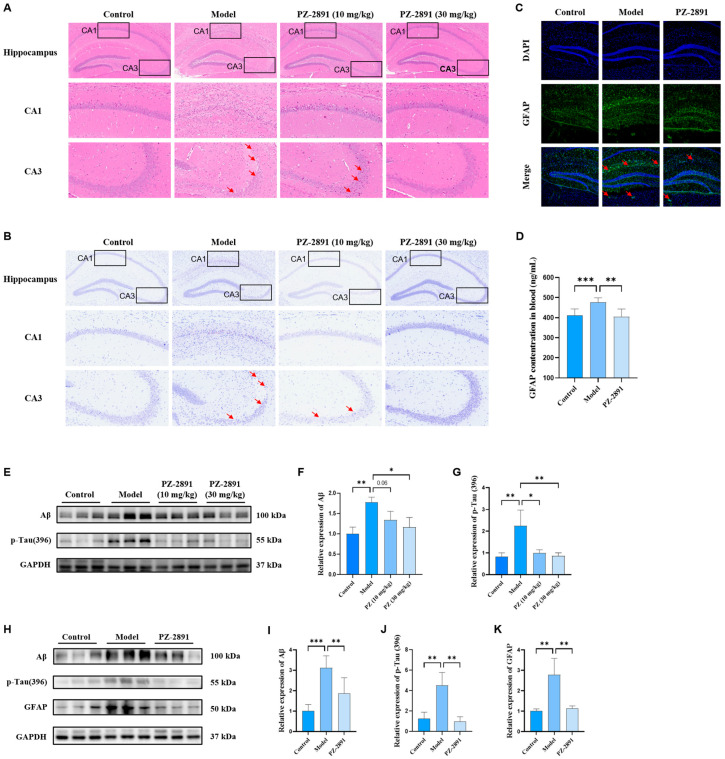
**PZ-2891** prevented neuronal damage and alleviated typical AD pathological characteristics both in vivo and in vitro. (**A**) HE staining results of control, model and treated mice, the arrows pointed to the damaged neurons. The magnification of the microscope is 8/20× (*n* = 3). (**B**) Nissl staining results for control, model and treated mice, the arrows pointed to the damaged neurons. The magnification of the microscope is 8/20× (*n* = 3). (**C**) IF images of GFAP in the hippocampus. The magnification of the microscope is 10× (*n* = 3). (**D**) GFAP expression in the blood (*n* = 6). (**E**) WB results for Aβ and p-tau in the hippocampus (*n* = 3). (**F**,**G**) Quantitative results of Aβ and p-tau expression (*n* = 3). (**H**) WB results of Aβ, p-tau and GFAP in HT22 cells induced by OA (*n* = 3). (**I**–**K**) Quantitative results of Aβ, p-tau and GFAP expression (*n* = 3). The data are expressed as the mean ± SD. *p* values were determined by one-way ANOVA. * *p* < 0.05, ** *p* < 0.01, *** *p* < 0.001.

HE staining revealed that neurons in the hippocampus of the model group were severely damaged, especially in the CA1 and CA3 regions (Figure 4A). Compared with the control group, the number of neurons in the model group decreased sharply, the arrangement of cells was disordered and cell shrinkage and inflammatory infiltration occurred. These conditions were restored by **PZ-2891** administration: the staining was clear and uniform, the number of neurons was restored and the cell arrangement was closer to that of the model group. Nissl staining revealed similar results in the model mice with severe neuronal degeneration. Reduced Nissl bodies and cell shrinkage were observed in the hippocampus, especially in the CA1 and CA3 regions. After the administration of **PZ-2891**, the morphology and arrangement of the neurons returned to their normal state, and the number of neurons and Nissl bodies normalized significantly (Figure 4B). Given the above findings, **PZ-2891** could prevent the neuronal damage to the hippocampus induced by Aβ_1-42_.

##### Biochemical Pathology

**Figure 5:**
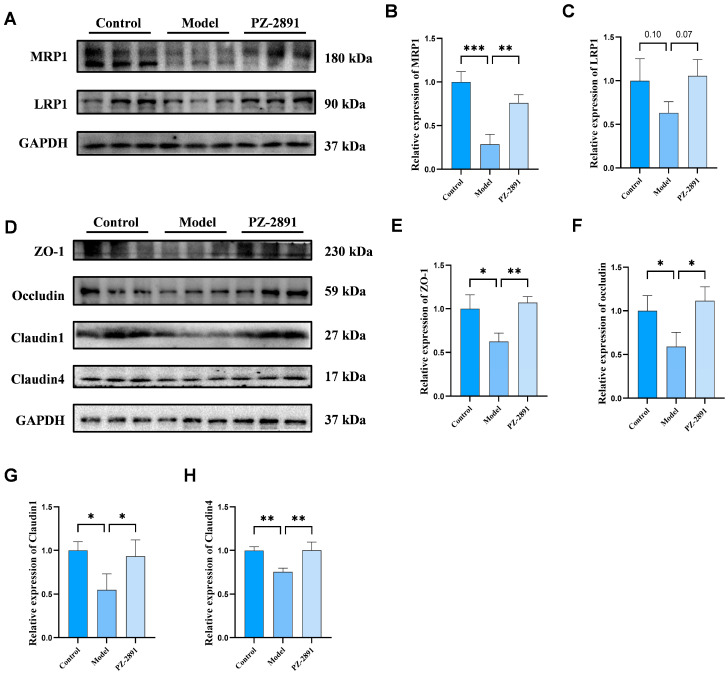
**PZ-2891** recovered the integrity and function of the BBB. (**A**) WB results of MRP1 and LRP1 expression in BMECs induced by OA (*n* = 3). (**B**,**C**) Quantitative results of MRP1 and LRP1 expression (*n* = 3). (**D**) WB results of ZO-1, occludin, claudin-1 and claudin-4 expression in BMECs (*n* = 3). (**E**–**H**) Quantitative results of ZO-1, occludin, claudin-1 and claudin-4 expression (*n* = 3). The data are expressed as the mean ± SD. *p* values were determined by one-way ANOVA. * *p* < 0.05, ** *p* < 0.01, *** *p* < 0.001.

As typical pathological characteristics of AD, the levels of Aβ and p-tau were determined through WB experiments (Figure 4E–G). The quantitative results revealed that the concentrations of Aβ and p-tau in the hippocampus of the model mice were greater than those in the control and treated groups, which is consistent with the results of the behavior test. These findings suggest that Aβ injection caused typical AD pathological features in C57 mice, whereas **PZ-2891** reduced Aβ and p-tau levels in a dose-dependent manner. GFAP is also considered a biomarker because of its positive association with AD incidence and development [23]. As shown in Figure 5D, the serum level of GFAP was decreased by **PZ-2891** treatment [24], suggesting it reduced serum biomarker expression in AD caused by Aβ_1-42_ invasion. The IF imaging results also revealed that the serum level of GFAP was elevated in the hippocampus of the model mice, whereas **PZ-2891** intervention decreased its expression (Figure 4C,D). Similarly, increased levels of Aβ, p-tau and GFAP were observed in vitro after OA incubation, the expression of which decreased significantly with **PZ-2891** treatment (Figure 4H–K). These findings indicate that **PZ-2891** intervention could effectively alleviate pathological symptoms of AD both in vivo and in vitro.

##### BBB Integrity and Transporter Expression

In addition to the above biomarkers, dysfunction of the blood–brain barrier (BBB), including impaired barrier integrity and abnormal expression of Aβ transporters, is closely related to the incidence and development of AD [25]. As illustrated in Figure 5D, the expression of tight junction (TJ) proteins, such as zonula occluden-1 (ZO-1), occludin, claudin-1 and claudin-4, clearly decreased in the model group. In addition, low-density lipoprotein receptor-related protein 1 (LRP1) and multidrug resistance-associated protein 1 (MRP1), which are responsible for the transport and clearance of Aβ, were also downregulated (Figure 5A–C) [26,27]. However, these proteins levels of brain microvascular endothelial cells were restored to normal after treatment with **PZ-2891**, suggesting that **PZ-2891** plays a positive role in improving the transportation function and integrity of the BBB.

#### 2.4.2. Positive Role of PANK2 in AD

**Figure 6:**
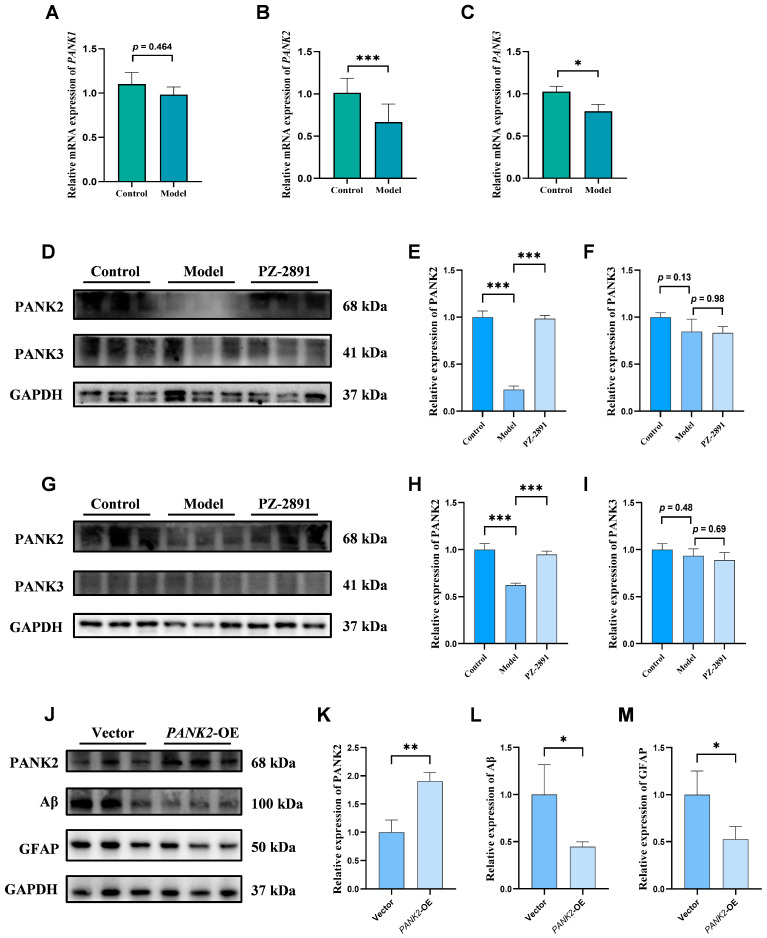
Compared with PANK1 and PANK3, **PZ-2891** more strongly influenced the modulation of PANK2, and PANK2 played a positive role in reducing AD pathologies. (**A**–**C**) Relative mRNA levels of three subtypes in the hippocampus (*n* = 8). (**D**–**F**) WB and quantitative results of PANK2 and PANK3 expression in vivo (*n* = 3). (**G**–**I**) WB and quantitative results of PANK2 and PANK3 expression in vitro (*n* = 3). (**J**) WB results for PANK2, Aβ and GFAP in the empty vector and *PANK2*-OE cell lines (*n* = 3). (**K**–**M**) Quantitative results of PANK2, Aβ and GFAP expression (*n* = 3). The data are expressed as the mean ± SD. *p* values were determined by unpaired Student’s t test for (**A**–**C**,**K**–**M**) and one-way ANOVA for (**E**,**F**,**H**,**I**). * *p* < 0.05, ** *p* < 0.01, *** *p* < 0.001.

Since **PZ-2891** was designed as a PANK modulator, its influence on three isoforms of the PANK gene was investigated in AD model mice. The PCR results revealed almost no effect on PANK1 expression, whereas the expression of PANK2 and PANK3 decreased in the hippocampus of the model mice (Figure 6A–C), especially PANK2, with a more significant difference.

The WB results revealed similar results. Compared with PANK3, **PZ-2891** significantly modulated PANK2 expression in both organ samples and cell samples (Figure 6D–I). These findings indicate that **PZ-2891** plays an important role in regulating the expression of PANK2, rather than that of PANK1 and PANK2, in AD.

Furthermore, to investigate the role of PANK2 in AD, a *PANK2*-OE cell line was established in this study. As shown in Figure 6J, compared with the empty vector, the upregulation of PANK2 contributed to decreased levels of Aβ and GFAP, both of which are AD pathological features, suggesting that PANK2 plays a positive role in AD treatment, while the mechanism through which PANK2 reduces AD biomarkers still requires further exploration.

### 2.5. Analytical Method Validation of PZ-2891

#### 2.5.1. Selectivity

**Figure 7:**
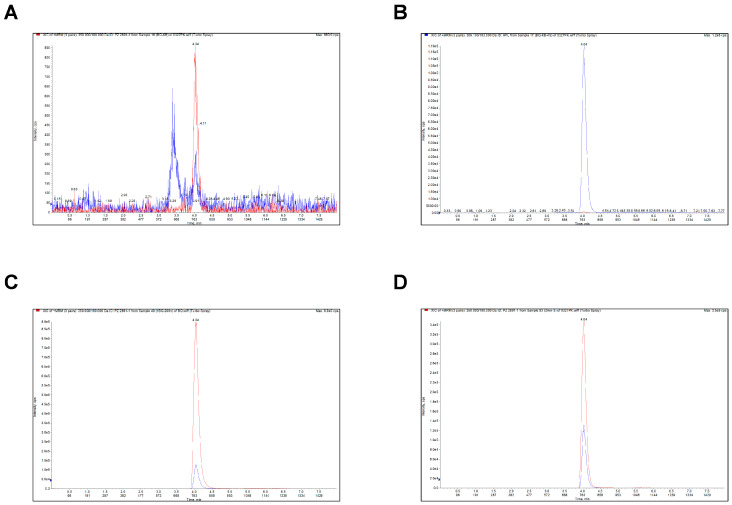
The established analytic method showed excellent selectivity and specificity. (**A**) The peak appearance of the blank plasma of SD rats. (**B**) The peak appearance of the blank plasma with 5 ng/mL IS. (**C**) The peak appearance of the blank plasma with 5 ng/mL IS and 200 ng/mL **PZ-2891**. (**D**) The peak appearance of the pre-treated plasma after oral administration of 7 mg/kg **PZ-2891** for 2 min. The blue peak represents warfarin (IS) and the red peak represents **PZ-2891**.

The multiple reaction monitoring (MRM) chromatogram results indicated that **PZ-2891** could be accurately distinguished from warfarin (internal standard, IS) and the endogenous components in the blank matrix, demonstrating that this analysis method showed excellent selectivity and specificity in both the blank samples and biological samples (Figure 7A–D).

#### 2.5.2. Linearity and Lower Limit of Quantitation (LLOQ)

The standard curve was obtained through linear regression calculations, with the final concentration of **PZ-2891** in plasma as the independent variable (x) and the ratio of the peak area (**PZ-2891**/IS) as the dependent variable (y). As shown in Table 1 and Supplementary Figure S2, the method showed excellent linearity (r^2^ > 0.998), precision (RSD < 5%) and accuracy (RE within ± 10%).

#### 2.5.3. Precision and Accuracy

As shown in Table 2, the intraday precision of **PZ-2891** was less than 7.99%, the inter-day precision was less than 5.51% and the accuracy ranged from 100.00 to 106.81%. The accuracy values, both intra-batch and inter-batch, were all within specified ranges, confirming that the method was reliable and sufficient for application in PK studies.

#### 2.5.4. Recovery and Matrix Effect

Quality control (QC) samples of three concentrations (5, 500 and 800 ng/mL) were used to evaluate the extraction recovery rate and matrix effect. The extraction recovery rate ranged from 96.65 to 103.68%, and the matrix effect ranged from 91.78 to 96.60%, confirming minimal interference (Supplementary Table S1).

#### 2.5.5. Stability

The QC samples remained stable under the following conditions: storage at room temperature for 12 h, cryopreservation at ultralow temperature for 7 days, placement in the autosampler for 24 h and three freeze–thaw cycles. All samples remained within ±10% deviation, indicating adequate stability under all tested conditions (Supplementary Table S2).

### 2.6. Blood Concentration–Time Curve of PZ-2891

Three groups were established to study the PK properties of **PZ-2891**: single low dose, single high dose and multiple high doses. In the single low dose group, after i.g. administration of **PZ-2891** (7 mg/kg), the peak drug concentration in the blood (C_max_) was 149.43 ± 53.29 ng/mL, the time to the peak concentration (T_max_) was 0.63 ± 0.14 h, the elimination half-life time (T_1/2_) was 1.56 ± 0.23 h and the area under the concentration–time curve (AUC_0-t_) was 284.41 ± 107.98 ng·h/mL (Table 3), suggesting rapid absorption and elimination in rats.

**Figure 8:**
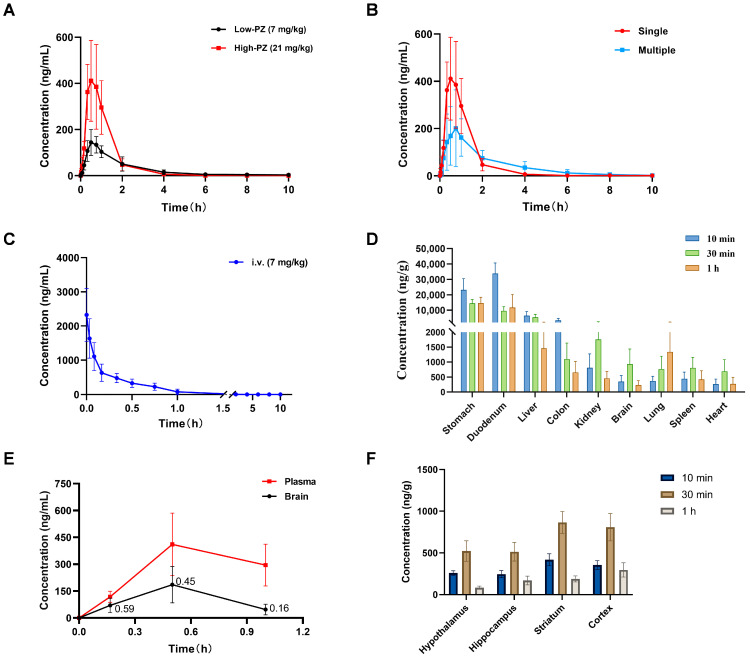
Blood concentration–time curves of **PZ-2891** under different conditions and distribution in tissues. (**A**) Curves of the single low dose group and single high dose group (*n* = 6). (**B**) Curves of the single high dose group and multiple high doses group (*n* = 6). (**C**) Blood concentration–time curve of the i.v. administration group (*n* = 6). (**D**) Distribution of **PZ-2891** in the main tissues: duodenum > stomach > liver > colon > kidney > brain > lung > spleen > heart; *p* < 0.05 (*n* = 6). (**E**) Plasma/brain concentration–time curves after i.g. administration of **PZ-2891** (*n* = 6). (**F**) Distribution of **PZ-2891** in the main regions of the brain: hypothalamus, hippocampus, striatum and cortex (*n* = 6). The data are expressed as the mean ± SD. *p* value was determined by one-way ANOVA.

The absorption and elimination patterns of the single high dose drug (21 mg/kg) were similar: plasma concentrations peaked at 0.5 h post-dose, followed by a mono-exponential decline (Figure 8A). The C_max_ of **PZ-2891** was 454.50 ± 151.35 ng/mL, and the AUC_0-t_ was 536.10 ± 184.01 ng·h/mL, which showed a dose-dependent pattern (Table 3), suggesting that a dose increase could increase exposure in rat blood. From the blood concentration–time curve, it could be speculated that **PZ-2891** has good oral bioavailability due to its rapid absorption, with a formulation optimization scope for its short half-life and predictable PK profiles for linear exposure.

In the multiple high doses group, blood samples were collected after i.g. administration of 21 mg/kg **PZ-2891** for 7 consecutive days. The C_max_ of **PZ-2891** was 208.58 ± 160.05 ng/mL, the T_max_ was 0.80 ± 0.21 h, the T_1/2_ was 1.05 ± 0.34 h and the AUC_0–t_ was 437.60 ± 167.23 ng·h/mL (Table 4). Compared with the single high dose, the time required to reach peak and elimination was longer, and the variation trends in absorption and elimination were relatively gradual (Figure 8B).

### 2.7. Oral Bioavailability Assessment of PZ-2891

After i.v. administration of 7 mg/kg **PZ-2891**, it entered the blood through the tail vein and quickly reached its peak concentration, and the C_max_ was 2330.00 ± 783.79 ng/mL (Figure 8C). The oral bioavailability of **PZ-2891** in SD rats was determined to be 19.74 ± 6.78% (Table 5), suggesting a scope for formulation enhancement, which was calculated by the equation F (%) = 100 × (D_i.v_ × AUC_i.g._)/(D_i.g._ × AUC_i.v._) [28]. In this equation, D_i.v._ represents the i.v. administration dose and D_i.g._ represents the i.g. administration dose.

### 2.8. Tissue Distribution of PZ-2891

As shown in Figure 8D and Supplementary Table S3, **PZ-2891** was widely distributed in various tissues, with concentrations ranging from high to low in the duodenum, stomach, liver, colon, kidney, brain, lung, spleen and heart. **PZ-2891** was relatively highly distributed in both the liver and kidney, indicating that it might be metabolized and eliminated through the liver and kidney. The stomach, duodenum, liver and colon showed similar tissue elimination kinetics: the concentration decreased gradually after 10 min. In addition to the lungs, the other tissues, including the kidney, brain, spleen and heart, exhibited a similar pattern: the drug concentration increased before 30 min and then decreased.

### 2.9. BBB Penetration Evaluation of PZ-2891

According to the tissue distribution results, considerable exposure to **PZ-2891** was observed in the brain, suggesting that it could cross the BBB and enter the brain. As shown in Figure 8E, similar to the blood curve, **PZ-2891** reached its peak concentration (185.83 ± 101.40 ng/mL) at approximately 30 min in the brain, while its elimination velocity was relatively faster than that of the blood. At 10 and 30 min, the concentration ratios (brain/blood) were 0.59 and 0.45, respectively, suggesting that **PZ-2891** can penetrate the BBB [29].

In addition to whole brain analysis, specific brain regions were also separated and analyzed. As shown in Figure 8F, the hypothalamus, hippocampus, striatum and cortex regions were considerably exposed, reaching peak concentrations of 523.25, 514.91, 866.67 and 809.58 ng/g, respectively, at approximately 30 min. These findings suggest that **PZ-2891** could not only penetrate the BBB but also be distributed in the hypothalamus, hippocampus, striatum and cortex, which is conducive to its therapeutic effect.

## 3. Discussion

AD is a prevalent neurodegenerative disorder with limited therapeutic methods. This study is the first to demonstrate that **PZ-2891**, a PANK modulator, plays a positive role in AD treatment.

In PD studies, **PZ-2891** has functioned as a cognitive improver in behavior experiments, with similar positive effects to donepezil. Notably, during the initial stage of dose exploration, mice treated with 10 mg/kg donepezil showed obvious salivation, tremors and muscle spasms, consistent with the reported peripheral side effects. Mice treated with **PZ-2891** still exhibited normal behavior at doses of 10 or 30 mg/kg, demonstrating a broader therapeutic window than donepezil. **PZ-2891** also acted as a neuroprotective agent both in vivo and in vitro: it not only restored neuronal morphology and reduced AD biomarkers of the damaged hippocampus but also increased the viability of cells invaded by D-gal and OA. The BBB is a natural dynamic barrier between the peripheral circulatory system and the central nervous system (CNS) and is dysfunctional under AD physiological conditions. **PZ-2891** exerted neuroprotective effects in restoring the integrity of the BBB and the expression of transporters responsible for removing Aβ, conducive to improving BBB function and clearing toxins. In addition, the secretion of proinflammatory cytokines (iNOS, COX-2, IL-6, IFN-γ, IL-1β and TNF-α) and oxidative stress were decreased significantly after coincubation with **PZ-2891**, which is beneficial for protecting the neural microenvironment, thereby alleviating pathological features and enhancing cognitive ability. **PZ-2891** was initially designed as a nonselective modulator of PANK, the rate-limiting enzyme for CoA biosynthesis, while the RT–qPCR and WB results revealed that it more strongly modulated PANK2 than other subtypes in AD pathology. The positive role of PANK2 was also revealed: PANK2 upregulation successfully decreased the levels of Aβ and GFAP. The dysfunction of CoA biosynthesis causes mitochondrial dysfunction, oxidative stress and iron metabolism imbalance, ultimately leading to neuronal degeneration. The intervention of **PZ-2891** might alleviate AD through PANK2 upregulation and CoA biosynthesis restoration.

According to PK investigation, **PZ-2891** exhibited favorable properties, including rapid absorption, wide distribution, avoidance of accumulation and ability to penetrate the BBB [30], with a brain-to-blood ratio of around 0.59, C_max_ of around 454.50 ± 151.35 ng/mL, AUC_0–t_ of around 536.10 ± 184.01 ng·h/mL and oral bioavailability of around 19.74 ± 6.78%. In particular, crossing the BBB has become one of the key evaluation criteria for anti-AD drug development [31,32]. **PZ-2891** exhibits advantages in administration dosage, oral bioavailability and BBB penetration over other emerging AD drugs. Notably, at the same dose (21 mg/kg), after multiple administrations, the C_max_ and AUC were both lower than the single administration. Unlike the dose-dependent pattern, exposure of **PZ-2891** did not occur in a time-dependent manner. Consecutive administration did not result in the accumulation of **PZ-2891**, thereby avoiding adverse reactions caused by drug accumulation. This phenomenon might be attributed to the following inferences: After multiple administrations, **PZ-2891** might induce increased expression and activity of drug-metabolizing enzymes in the liver, kidney or other tissues, thereby accelerating the metabolic process of the drug. Consecutive administration might affect the function of transporters, and changes in transporter expression or activity might possibly influence the absorption, distribution and efflux of the drug. The rapid clearance might also be attributed to metabolic induction and transporter regulation. Taken together, these findings indicate that **PZ-2891** is a new indicator for AD. This potential candidate has excellent efficacy and PK properties and deserves further investigation.

Although we revealed the positive role of **PZ-2891** in AD and its PK properties, this study has several limitations. First, the animal model was established based on Aβ pathology, and incorporating a model of tau phosphorylation could strengthen the persuasiveness of the therapeutic effects of **PZ-2891** in vivo. Second, the in vivo research was conducted on male mice or rats; further studies should include female mice and rats since sex bias is not negligible in AD investigation. Third, this compound could not be detected after 10 h in SD rats. Although this avoided the accumulation of toxicity, it also indicates rapid drug elimination. Thus, further research on structural modification and optimization based on **PZ-2891** should be carried out to obtain better druggability and prolonged elimination. The isopropyl group of **PZ-2891** is prone to oxidative metabolism, so it could be replaced by a more stable group, such as a cyclopropyl group. A methyl group could also be added to the piperazine to alleviate its molecular planarity and crystalline packing. A self-microemulsion drug delivery system (SMEDDS) or lipid nano-hydrate suspension could be imported to improve its diffusion and absorption, thereby extending the duration of effective exertion.

## 4. Materials and Methods

### 4.1. Chemicals and Reagents

The reagents and chemicals were shown in Table 6.

### 4.2. Animal Model Establishment

#### 4.2.1. Animals

Eight-week-old male C57BL/6J mice were purchased from Vital River Laboratories (Beijing, China). The mice were maintained under specific pathogen-free (SPF) conditions with a 12 h light/dark cycle in a thermally regulated environment, and were provided ad libitum access to food and water. All experimental protocols were approved by the Animal Ethics Committee of China Pharmaceutical University (approval no.: 2024-09-007; approval date: September 2024). Following adaptive feeding, the mice were randomly divided into five groups (control, model, positive drug, low dose and high dose; n = 8 per group) using a computer-generated random number table. The randomization code was held by an independent technician who was not involved in the subsequent procedures. The experimenters performing behavioral assessments were blinded to the group identities.

#### 4.2.2. Aβ_1-42_ Injection

Aβ_1-42_ peptide powder was dissolved in 1,1,1,3,3,3-hexafluoroisopropanol (HFIP); then, the solution was volatilized to form a transparent peptide membrane. The peptide was redissolved by sterile saline to obtain a 4 mg/mL solution, which was incubated at 37 °C for 5 days to promote oligomerization (Supplementary Figure S3) [33]. The mice were injected through the intracerebral ventricle (icv.) with Aβ solution after anesthesia (10 µg per mouse). The injection site was ML 1.95 mm, AP −2.3 mm, DV −1.7 mm according to the mouse brain in stereotaxic coordinates, and the injection volume was 2.5 µL. The control mice were injected at the same volume with normal saline. The mice injected with Aβ were divided to four groups: model, donepezil (5 mg/kg), **PZ-2891** (10 mg/kg) and **PZ-2891** (30 mg/kg). The oral administration duration lasted for two weeks.

### 4.3. Behavioral Experiments

#### 4.3.1. Morris Water Maze (MWM)

The platform (radius: 5 cm) was placed in the fourth quadrant of the circular pool (height: 45 cm; radius: 60 cm). The water level was 1~2 cm higher than the platform, and the temperature was maintained at 22 ± 1 °C. Experiments were conducted in a quiet environment to avoid the influence of sound and light. During the training period, mice were placed in the pool from random quadrants. Their swimming trajectory and escape latency were recorded. If the mice could not find the target platform within 60 s, guidance would be provided to enhance their memory. During the searching period, the objective platform was removed, and the number of mice crossing this area was recorded to evaluate the memory and study ability of the mice.

#### 4.3.2. Open Field Test (OFT)

The OFT was conducted to evaluate the locomotor activity and exploratory behavior of AD mice. An open box (length: 50 cm; width: 50 cm; height: 40 cm), connected to the camera, was set to provide a wide place for mouse autonomous movement. After adaptation, the mice were put into the box, and significant parameters were recorded within 5 min, including the movement trajectory, speed and duration in the center. Experiments were conducted in a quiet and bright environment. Afterwards, 75% ethanol was used to clean the traces and avoid odors left by the previous mouse. Anxious mice tended to move around the peripheral area and showed reduced exploration in the central area for their thigmotaxis. The total movement distance and speed could be used to assess their athletic ability.

#### 4.3.3. Novel Object Recognition (NOR)

Two identical objects were placed in the open field, and the exploration condition of the mouse on each object was recorded within 5 min. One hour later, a new object was used as a substitute, and the trajectory of exploring this novel object was recorded and measured within 5 min. The recognition index (RI) = (new object)/(new object + old object) × 100%.

#### 4.3.4. Y Maze

The mouse was placed at the end of one arm to explore in the Y maze freely for 5 min. The trajectory and number of times each arm was entered were recorded. The alternation percentage = (total number of alternations)/(maximum number of alternations) × 100%. During the new arm recognition test, one arm was closed and the mouse could move freely in the other two arms. One hour later, the closed arm was recovered as a new arm, and the trajectory of exploring the Y maze was recorded within 5 min. The new arm recognition rate = (new arm)/(new arm + old arms) × 100%. The software used for behavioral data analysis was ANY-maze 6.0.

### 4.4. Cell Culture and CCK-8 Assay

HT22, PC12 and BV2 cells were all purchased from the Cell Culture Center of the Chinese Academy of Medical Sciences. All cell lines were cultured in a medium consisting of 90% DMEM and 10% FBS at 37 °C and 5% CO_2_. Cells were incubated in 96-well plates (8000/well) for 24 h, then D-gal (15 mg/mL) and OA (40 nM) were added to cells to cause cell damage and death, and **PZ-2891** (0.5, 1, 2 µM) was added to improve the viability. Twenty-four hours later, a CCK-8 solution (10%) was added to the wells and incubation took place for 1 h. Absorbance was measured at 450 nm using a Synergy H1 Microplate Reader. The viability = (As − Ab)/(Ac − Ab) × 100% (As: experimental well absorbance; Ac: control well absorbance; Ab: blank well absorbance). All the cell experiments were repeated at least three times.

### 4.5. Reactive Oxygen Species (ROS) Assay

The level of intracellular ROS was reflected by the fluorescence intensity of DCF (2′,7′-Dichlorofluorescein). PC12 cells were incubated in 96-well plates (1 × 10^5^/well) at 37 °C for 24 h. Then, the cells were treated with LPS (1 µg/mL) and **PZ-2891** (0.25, 0.5 µM) overnight [34]. DCFH-DA (2′,7′-Dichlorodihydrofluorescein diacetate) solution was added to the cells (500 µL/well). After incubation for 30 min at 37 °C, the cells were washed with PBS three times to remove residual DCFH-DA. The excitation wavelength of 488 nm and emission wavelength of 525 nm were set to detect the intensity of fluorescence using a BioTek Living Cell Analysis System.

### 4.6. Reverse Transcription Quantitative Polymerase Chain Reaction (RT-qPCR)

The RNA extraction was obtained with Trizol, and cDNA was acquired through reverse transcription with RT SuperMix reagent (Vazyme Biotech Co., Ltd., Nanjing, China). The RT-qPCR assay was performed on a CFX96 Real-Time PCR cycler (Bio-Rad, Hercules, CA, USA). The relative mRNA expression was determined via a comparative 2^−ΔΔCT^ method. The primers were synthesized by Sangon Biotech Co., Ltd. (Shanghai, China). GAPDH: (forward: 5′-AGGTCGGTGTGAACGGATTTG-3′; reverse: 5′-TGTAGACCATGTAGTTGAGGTCA-3′); PANK1: (forward: 5′-GTTCGCCCAGCATGATTCTC-3′; reverse: 5′-CTTAACCAGGGTTCCACCGAT-3′); PANK2: (forward: 5′-GAGGCGGAGAGTGTGAGAC-3′; reverse: 5′-CCAAGGTTCCTCCAATATCCAAG-3′); PANK3: (forward: 5′-GGACATTGGAGGAACGCTAGT-3′; reverse: 5′-ACATCCCGAATGCCAGTAGAT-3′); COX2: (forward: 5′-TGCACTATGGTTACAAAAGCTGG-3′; reverse: 5′-TCAGGAAGCTCCTTATTTCCCTT-3′); iNOS: (forward: 5′-GTTCTCAGCCCAACAATACAAGA-3′; reverse: 5′-GTGGACGGGTCGATGTCAC-3′); IFN-γ: (forward: 5′-GGAGGAACTGGCAAAAGGATG-3′; reverse: 5′-GTTGCTGATGGCCTGATTGT-3′); IL-1β: (forward: 5′-GCAACTGTTCCTGAACTCAACT-3′; reverse: 5′-ATCTTTTGGGGTCCGTCAACT-3′); IL-6: (forward: 5′-ACAAGTCGGAGGCTTAATTACACAT-3′; reverse: 5′-TTGCCATTGCACAACTCTTTTC-3′); TNF-α: (forward: 5′-GACCCTCACACTCAGATCATCTT-3′; reverse: 5′-CCTTGAAGAGAACCTGGGAGTAG-3′).

### 4.7. Western Blot (WB) Analysis

RIPA lysis buffer (with 1% protease inhibitor and 1% phosphatase inhibitor) was used to obtain protein samples from cells and tissues. SDS-PAGE was used to separate the protein samples in the Tris-glycine electrophoresis system. After transferring to a PVDF membrane and blocking in 5% BSA or skim milk for 2 h, blots were incubated with primary antibodies at 4 °C overnight, namely GAPDH (1:40,000, Abcam EPR16891), p-tau396 (1:500, CST F3S9T), Aβ (1:1000, Proteintech 25524-1-AP), GFAP (1:10,000, Proteintech 16825-1-AP), LRP1 (1:1000, Proteintech 26106-1-AP), MRP1 (1:1000, Proteintech 67228-1-Ig), PANK1 (1:1000, Proteintech 11768-1-AP), PANK2 (1:1000, Proteintech 11001-1-AP), PANK3 (1:500, SCBT sc-551231), occludin (1:2000, Proteintech 27260-1-AP), ZO-1 (1:1000, Proteintech 21773-1-AP), claudin-1 (1:1000, Proteintech 28674-1-AP) and claudin-4 (1:1000, Proteintech 16195-1-AP). After incubating with secondary antibodies (1:10,000 Bioworld BS13278) for 2 h, the target proteins were detected in a ChemDocTM XRS+ gel imaging system via the enhanced chemiluminescence (ECL) method.

### 4.8. Hematoxylin–Eosin (HE) Staining

The brain tissue was extracted from the fixative (4% paraformaldehyde), dehydrated and embedded in paraffin wax. The cell nucleus was stained purple by an alkaline hematoxylin solution and the cytoplasm was stained red by an acidic eosin solution. The HE staining slide was observed using a panoramic tissue section scanner (NanoZoomer S60, Hamamatsu, Japan).

### 4.9. Nissl Staining

In histology, Nissl bodies function as a sensitive indicator of nerve cell damage. Paraffin sections and the observation method were the same as 2.8. The alkaline toluidine blue solution (0.5%) was used to make the Nissl bodies appear dark blue and the cell nuclei appear light blue. Nissl staining reflects the pathological morphology of nerve cells.

### 4.10. Immunofluorescence (IF) Assay

The mouse brain was fixed with 4% paraformaldehyde and embedded by OCT after dehydration. Brain sections (14 µm) were obtained by freezing microtome and permeabilized with 0.5% Triton X-100 for 30 min, then blocked in 5% BSA solution. Sections were incubated in the primary antibody (anti-GFAP antibody, 1:6000) at 4 °C for 24 h, which was diluted in 3% BSA. After incubating in the secondary antibody (anti-rabbit IgG-FITC), DAPI was applied to the cell nucleus (Beyotime C1005). The final images were obtained through a laser scanning confocal microscope (Olympus FV3000, Tokyo, Japan) and quantitative results were analyzed using Image J (version 1.53k).

### 4.11. Liquid Chromatography–Tandem Mass Spectrometry (LC-MS/MS) Conditions

A triple quadrupole liquid chromatography–mass spectrometry instrument (SCIEX Triple Quad 5500+, Tokyo, Japan) was used to conduct **PZ-2891** concentration determination. It was analyzed using the electro spray ionization (ESI) interface in the positive ion mode. The ESI source parameters were as follows (Table 7): ion-spray voltage (IS) 5500 V, ion source temperature (TEM) 550 °C and collision gas (CAD) 10 Psi. The MRM parameters of **PZ-2891** were as follows: Q1: 350 Da; Q3: 190 and 133 Da; DP: 16 V; and CE: 25 and 41 V. The MRM parameters of warfarin were as follows: Q1: 309 Da; Q3: 163 Da; DP: 65 V; and CE: 30 V.

As shown in Table 7, the chromatographic column was ZORBAX Eclipse Plus C18 (2.1 × 50 mm, 3.5-Micron). The column temperature was set at 40 °C and the samples were set at 4 °C. The mobile phase A (aqueous phase) was ultrapure water with 0.1% formic acid and 5 mM formic acid ammonium, and the mobile phase B (organic phase) was pure acetonitrile. The gradient program was the following: 0.0–1.0 min, 20% B; 1.0–2.5 min, 20–90% B; 2.5–6.0 min, 90% B; 6.0–7.0 min, 90–20% B; and 7.0–8.0 min, 20% B.

### 4.12. Preparation of Standard Samples

Warfarin was employed as the internal standard (IS) substance for its stability and similar response characteristics to the test compound. The retention times of the internal standard and **PZ-2891** were both 4.04 min. The mass spectrometer was tuned and calibrated in three steps: (1) ion source and transmission optimization, (2) MS calibration and (3) fine tuning (detection maximization of one or more particular ions). A 1 mg/mL **PZ-2891** and warfarin solution was prepared and stored at −20 °C. The protein precipitation agent was obtained from diluting the warfarin solution, and the final concentration was 5 ng/mL. A series of gradient working solutions of **PZ-2891** ranging from 20 to 10,000 ng/mL was prepared, and 5 µL of the working solution was added into 45 µL blank rat plasma to obtain standard curve samples with concentrations of 2, 5, 10, 20, 50, 100, 200, 500 and 1000 ng/mL. The r^2^ value of the three calibration curves was 0.9987.

### 4.13. Method Validation

Method validation was composed of selectivity, standard curve and quantitative limit, precision and accuracy, matrix effect, extraction recovery rate and stability verification. All protocols complied with FDA (2018) and EMA (2022) bioanalytical guidance.

### 4.14. Pharmacokinetic (PK) Study

Sprague Dawley (SD) rats (male, 6–8 weeks, 200 ± 20 g) were purchased from Vital River Laboratory Animal Technology Co., Ltd. (Beijing, China). Before the experiment, all rats were fasted overnight for 8–12 h with free access to water. The SD rats underwent intragastric (i.g.) administration of 7 or 21 mg/kg **PZ-2891** and intravenous (i.v.) administration at a dose of 7 mg/kg. Blood samples were collected from the orbital venous plexus at 2 min, 5 min, 10 min, 20 min, 30 min, 45 min, 1 h, 2 h, 4 h, 6 h, 8 h and 10 h after administration, and were centrifuged at 8000 rpm to obtain a supernatant. Tissue samples were collected at 10 min, 30 min and 1 h and stored at −80 °C.

### 4.15. Preparation of Biological Samples

A total of 50 µL SD rat plasma was added into 250 µL protein precipitation agent, and then shook at 1800 rpm for 5 min. After centrifugation at 18,000 rpm twice, 5 µL of the solution was injected for LC-MS/MS analysis. Tissues, including duodenum, stomach, liver, colon, kidney, brain, lung, spleen and heart tissues, were collected to determine the concentration. Tissue samples were accurately weighed to 100 mg and cut into pieces. Three zirconium beads and 500 µL of saline were added to prepare a 200 mg/mL tissue homogenate solution without obvious visible tissue fragments, and subsequent protocols were the same as the blood samples.

### 4.16. Statistical Analysis

The pharmacokinetic parameters of **PZ-2891** in SD rats were calculated using the non-compartmental (NCA) model in WinNonlin 8.1 software (Certara, Princeton, CA, USA). Quantitative data were presented as the mean value ± standard deviation (SD), and the significance threshold (*p* value) was less than 0.05. An unpaired Student’s *t*-test and normality testing were utilized to compare two independent groups, and one-way ANOVA and normality testing were used for more than two independent groups using GraphPad Prism 9.0 software.

## 5. Conclusions

Taken together, this study discovered **PZ-2891** as a potential anti-AD compound through behavioral tests and neuroprotective experiments. In vivo and in vitro data revealed that **PZ-2891** significantly alleviated cognitive impairment and neuroinflammation and reduced typical AD pathological markers, including p-tau, Aβ and GFAP. It was also confirmed that the upregulation of PANK2 expression positively alleviated pathologies, indicating its active role in AD treatment. In addition, **PZ-2891** exhibited favorable PK properties, with a brain-to-blood ratio of around 0.59, C_max_ of around 454.50 ± 151.35 ng/mL, T_max_ of around 0.49 ± 0.15 h and oral bioavailability of around 19.74 ± 6.78%. It could disperse rapidly in the blood and main tissues without drug accumulation, and its capability of crossing the BBB was also conductive to exerting therapeutic efficacy in the CNS. In conclusion, **PZ-2891** is worthy of further anti-AD investigation, and its PK properties also provide valuable references for the optimization of derivatives.

## Figures and Tables

**Table 1:** The regression equations, linear ranges and LLOQs for the determination of the analyte in rat plasma.

Compound	Regression Equation	R	Linear Range (ng/mL)	LLOQ (*n* = 5)		
				Measured Concentration	RSD (%)	RE (%)
**PZ-2891**	y = 0.0264x + 0.0108	0.9987	2–1000	2.11	4.34	5.50

**Table 2:** Precision and accuracy for the analyte in rat plasma.

Compound Spiked Concentration (ng/mL)	Intra-Day (*n* = 5)			Inter-Day (*n* = 5)		
	Measured Concentration (ng/mL)	Precision (RSD, %)	Accuracy (RE, %)	Measured Concentration (ng/mL)	Precision (RSD, %)	Accuracy (RE, %)
2	2.05 ± 0.16	7.99	2.50	2.04 ± 0.11	5.51	1.77
5	5.13 ± 0.11	2.08	2.56	5.28 ± 0.17	3.31	5.51
500	524.40 ± 15.47	2.95	4.88	534.07 ± 18.07	3.38	6.81
800	809.60 ± 7.80	0.96	1.20	823.13 ± 15.31	1.86	2.89

**Table 3:** PK parameters in rat plasma after a single low dose (7 mg/kg) or a single high dose (21 mg/kg) of **PZ-2891** (means ± SDs, *n* = 6).

Dose (mg/kg)	T_1/2_ (h)	T_max_ (h)	C_max_ (ng/mL)	AUC_0–t_ (ng∙h/mL)	AUC_0–∞_ (ng∙h/mL)	V/F (L/kg)	CL/F (L/h/kg)	MRT (h)
7	1.56 ± 0.23	0.63 ± 0.14	149.43 ± 53.29	284.41 ± 107.98	288.24 ± 109.16	61.96 ± 25.27	27.45 ± 10.35	2.00 ± 0.26
21	0.94 ± 0.23	0.49 ± 0.15	454.50 ± 151.35	536.10 ± 184.01	536.60 ± 184.02	55.10 ± 29.04	44.24 ± 19.32	1.05 ± 0.23

**Table 4:** PK parameters of **PZ-2891** in rat plasma after oral administration of 21 mg/kg **PZ-2891** for one day or seven days (means ± SDs, *n* = 6).

i.g. (Days)	T_1/2_ (h)	T_max_ (h)	C_max_ (ng/mL)	AUC_0–t_ (ng∙h/mL)	AUC_0–∞_ (ng∙h/mL)	V/F (L/kg)	CL/F (L/h/kg)	MRT (h)
1	0.94 ± 0.23	0.49 ± 0.15	454.50 ± 151.35	536.10 ± 184.01	536.60 ± 184.02	55.10 ± 29.04	44.24 ± 19.32	1.05 ± 0.23
7	1.05 ± 0.34	0.80 ± 0.21	208.58 ± 160.05	437.60 ± 167.23	439.99 ± 168.25	409.48 ± 323.92	218.04 ± 128.00	2.17 ± 0.91

**Table 5:** Oral bioavailability assessment of **PZ-2891** in rat plasma (means ± SDs, *n* = 6).

Route	Dose (mg/kg)	T_1/2_ (h)	T_max_ (h)	C_max_ (ng/mL)	AUC_0–t_ (ng∙h/mL)	V/F (L/kg)	CL/F (L/h/kg)	Oral Bioavailability (%)
i.g.	21	0.94 ± 0.23	0.49 ± 0.15	454.50 ± 151.35	536.10 ± 184.01	55.10 ± 29.04	44.24 ± 19.32	19.74 ± 6.78
i.v.	7	0.52 ± 0.20	0.03 ± 0.00	2323.33 ± 777.81	905.32 ± 222.80	4.19 ± 3.51	5.77 ± 5.07	

**Table 6:** Chemicals and reagents.

Reagents	Catalog Numbers	Purity	Supplier
Warfarin	13566	>98%	Cayman Chemical, Ann Arbor, MI, USA
Acetonitrile	34888	>99.9%	Merck KGaA, Darmstadt, Germany
Methanol	34860	>99.9%	Merck KGaA, Darmstadt, Germany
Formic acid	28905	>99%	Thermo Fisher Scientific, Waltham, MA, USA
**PZ-2891**	HY-124634	99.68%	Med Chem Express, Monmouth Junction, NJ, USA
DMEM	D0822	-	Sigma-Aldrich, St. Louis, MO, USA
FBS	12103C	-	Sigma-Aldrich, St. Louis, MO, USA
Amyloid β Peptide	P9001	>95%	Beyotime, Shanghai, China
RIPA lysis buffer	P0013E	-	Beyotime, Shanghai, China
Cell Counting Kit-8	C0039	-	Beyotime, Shanghai, China
ALT	C009-2-1	-	Jiancheng, Nanjing, China
AST	C010-2-1	-	Jiancheng, Nanjing, China
BUN	C013-1-1	-	Jiancheng, Nanjing, China
CRE	C011-2-1	-	Jiancheng, Nanjing, China

**Table 7:** Analyte-specific LC–MS/MS parameters.

	Instrument Variables	
LC	Chromatographic column	ZORBAX Eclipse PlusC18 (2.1 × 50 mm 3.5-Micron)
	Injection volume	5 μL
	Column temperature	40 °C
	Flow rate	0.15 mL/min
	Mobile phase	(A) 0.1% formic acid in water + 5 mM ammonium formate; (B) Acetonitrile
MS/MS	MRM transition, *m*/*z*	350 → 190
	Declustering potential, V	16
	Entrance potential, V	10
	Collision energy, V	25
	Collision cell exit potential, V	10

## Data Availability

The original contributions presented in this study are included in the article/Supplementary Material. Further inquiries can be directed to the corresponding authors.

## Supplementary Materials

The following supporting information can be downloaded at https://www.mdpi.com/article/10.3390/ph18121871/s1, Figure S1: **PZ-2891** showed good oral safety at a dosage of 30 mg/kg; Figure S2: The linear standard curve of **PZ-2891**, with the correlation coefficient (r) surpassing 0.998; Table S1: Matrix effect and extraction recovery for the analyte in rat plasma; Table S2: Stability of the analyte in rat plasma; Table S3: The concentration values of rat tissues (mean ± SD, n = 6); Figure S3: Aβ_1–42_ oligomerization determination.

## Abbreviations

The following abbreviations are used in this manuscript:

AD	Alzheimer’s disease
ADI	Alzheimer’s Disease International
ARIA-E	amyloid-related imaging abnormalities—edema
ARIA-H	amyloid-related imaging abnormalities—hemorrhage
Aβ	amyloid-β
AUC	area under the concentration–time curve
BBB	blood–brain barrier
BUN	blood urea nitrogen
CCK8	cell counting kit 8
CoA	coenzyme A
COX-2	cyclooxygenase-2
CNS	central nervous system
CRE	creatinine
DCF	2′,7′-Dichlorofluorescein
DCFH-DA	2′,7′-Dichlorodihydrofluorescein diacetate
DMEM	Dulbecco’s modified Eagle’s medium
D-gal	D-galactose
FDA	Food and Drug Administration
GFAP	glial fibrillary acidic protein
GFI	green fluorescence intensity
IFN-γ	interferon-γ
IL-1β	interleukin-1β
IL-6	interleukin-6
iNOS	inducible nitric oxide synthase
IS	internal standard
LC–MS/MS	liquid chromatography–mass spectrometry
LLOQ	lower limit of quantitation
LPS	lipopolysaccharide
LRP1	low-density lipoprotein receptor-related protein 1
MRM	multiple reaction monitoring
MRP1	multidrug resistance-associated protein 1
MWM	Morris water maze
NCA	non-compartmental
NOR	novel object recognition
OFT	open field test
OA	okadaic acid
PD	pharmacodynamic
PK	pharmacokinetic
PKAN	pantothenate kinase-associated neurodegeneration
PANK	pantothenate kinase
QC	quality control
RE	relative deviation
SD	Sprague Dawley
TJ	tight junction
TNF-α	tumor necrosis factor-α
ZO-1	zonula occluden-1
