# Supplementary material for: Pharmacokinetic and Pharmacodynamic Evaluation of PZ-2891, an Anti-Alzheimer’s Disease Agonist of PANK2
Source: Pharmaceuticals (Basel). 2025 Dec 9;18(12):1871. doi: 10.3390/ph18121871 (PMC12735420; doi:10.3390/ph18121871)
Supplement: Supplementary file 1 [file pharmaceuticals-18-01871-s001.zip › pharmaceuticals-3987221-supplementary.pdf]

Supplementary Materials

Pharmacokinetic and pharmacodynamic evaluation of PZ-2891, an anti-Alzheimer's disease agonist of PANK2

Ying Chen, Huimin Ma, Mengyao Jin, Shize Zhang, Shimeng Qu, Guangji Wang*, Jiye Aa*

Key Laboratory of Drug Metabolism and Pharmacokinetics, State Key Laboratory of Natural Medicines, China Pharmaceutical University, Nanjing, PR China

These authors contributed equally to this work.

* *Corresponding authors at: Gulou District, Tongjia Lane 24, Nanjing 210009, PR China. E-mail addresses: gjwang@cpu.edu.cn (G.Wang), jiyea@cpu.edu.cn (J.Aa)*

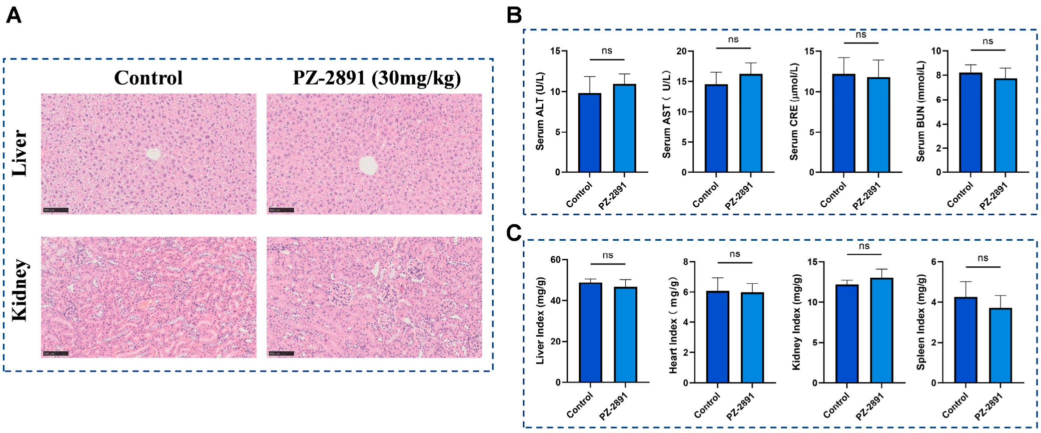

Figure S1. PZ-2891 showed good oral safety at a dosage of 30 mg/kg. (A) No obvious damage was observed in the HE staining of the blank and **PZ-2891**-administered mice. (B) No significant difference in the levels of ALT, AST, BUN and CRE was detected between the control and **PZ-2891**-treated mice. (C) No significant difference was observed in the organ indices of the control and **PZ-2891**-treated mice. ns: not significant.

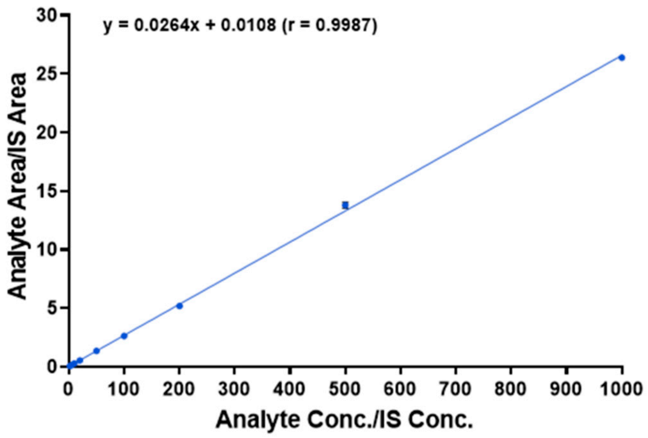

Figure S2. The linear standard curve of **PZ-2891**, with the correlation coefficient (r) surpassing 0.998.

Table S1. Matrix effect and extraction recovery for the analyte in rat plasma. [a]

Compound spiked concentration (ng/mL)	Matrix effect (*n*=6)		Extraction recovery (*n*=6)	
	Mean (%)	RSD, (%)	Mean (%)	RSD, (%)
5	91.78	5.44	96.65	9.05
500	92.23	2.48	103.68	3.35
800	96.60	9.57	101.99	7.84

[a] The extraction recovery was determined by comparing the peak areas of analytes and internal standard (IS) in quality control (QC) samples (5, 500, and 800 ng/mL) with those of analytes and IS spiked into post-extraction blank plasma. The matrix effect was evaluated by comparing the peak areas of analytes and IS spiked into post-extraction blank plasma with those of pure standard solutions.

Table S2. Stability of the analyte in rat plasma. [a]

Compound spiked concentration (ng/mL)	Short-term stability (room temperature for 12h)		Long-term stability (−80°C for 7d)		Free-thaw stability (3 freethaw cycles)		Post-preparation stability (4°C for 24 h)	
	RSD (%)	RE (%)	RSD (%)	RE (%)	RSD (%)	RE (%)	RSD (%)	RE (%)
5	2.75	-1.04	6.97	0.84	1.87	-0.28	6.95	-1.52
500	2.96	-0.48	2.16	8.64	1.08	7.20	0.74	3.72
800	2.03	-2.72	6.78	6.93	3.09	5.55	1.77	-0.20

[a] The stability of the analyte was evaluated by quality control (QC) samples at three concentration levels (5, 500, and 800 ng/mL). Short-term stability was assessed by placing the processed samples at room temperature for 12 hours. Long-term stability was determined by storing the samples at −80 °C for 7 days. Autosampler stability was investigated by maintaining the samples in the autosampler chamber for 24 hours. Freeze-thaw cycle stability was analyzed after the samples underwent three freeze (−80 °C) and thaw cycles. The results showed that the relative error (RE)

under all the aforementioned conditions was within ±10%.

Table S3. The concentration values of rat tissues (mean ± SD, n = 6).

Tissue	10min		30min		1h	
	Mean	SD	Mean	SD	Mean	SD
Stomach	23150.00	7353.43	14466.67	2507.72	14591.67	3838.28
Duodenum	33808.33	6801.87	9561.67	2735.40	11753.33	8520.88
Liver	6550.00	2597.27	5446.67	1899.96	1463.33	830.64
Colon	3395.00	1271.87	1099.50	537.68	658.42	365.87
Kidney	807.83	467.43	1761.67	793.37	460.08	231.97
Brain	350.50	205.22	929.17	507.01	235.33	147.75
Lung	371.33	155.88	772.58	423.42	1338.33	1057.56
Spleen	443.00	226.57	805.00	350.41	423.17	291.96
Heart	267.58	170.24	692.75	388.67	272.83	222.28

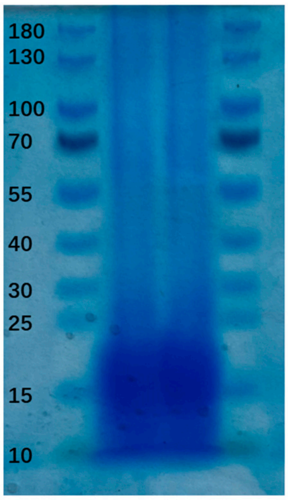

Figure S3. A$\beta_{1\text{-}42}$ oligomerization determination.

Figure 3L

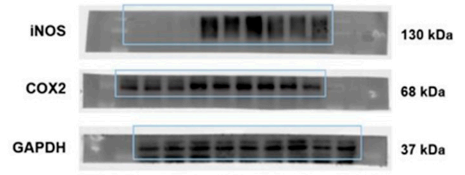

Figure 4E

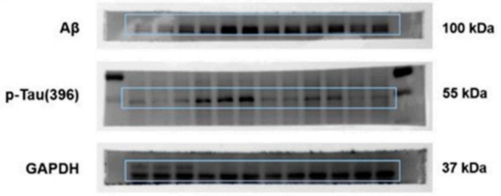

Figure 4H

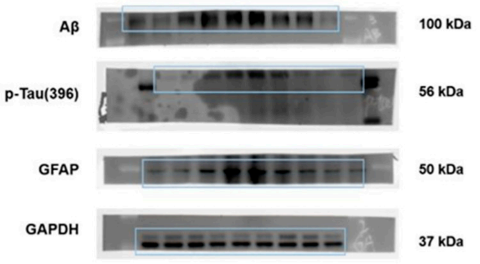

Figure 5A

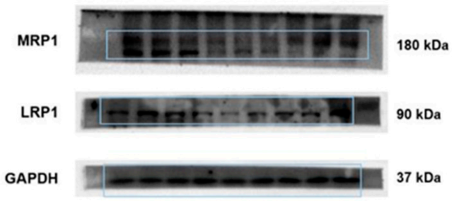

Figure 5D

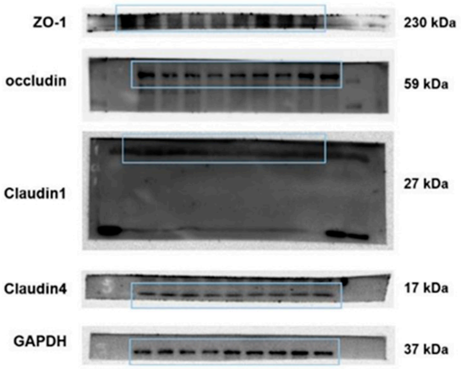

Figure 6D

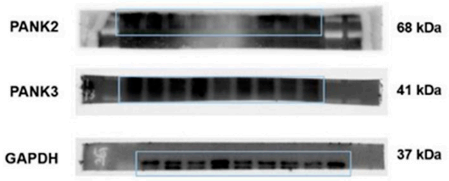

Figure 6G

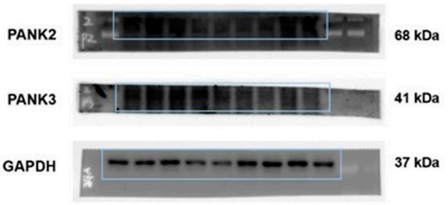

Figure 6J

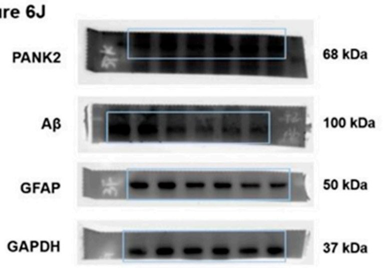

uncropped blots
